# Inferring SARS-CoV-2 RNA shedding into wastewater relative to the time of infection

**DOI:** 10.1017/S0950268821002752

**Published:** 2022-01-07

**Authors:** Sean Cavany, Aaron Bivins, Zhenyu Wu, Devin North, Kyle Bibby, T. Alex Perkins

**Affiliations:** 1 Department of Biological Sciences, University of Notre Dame, Notre Dame, USA; 2 Department of Civil and Environmental Engineering and Earth Sciences, University of Notre Dame, Notre Dame, USA

**Keywords:** SARS-CoV-2, COVID-19, virus shedding, waste water, environmental monitoring, wastewater-based epidemiological monitoring, public health surveillance

## Abstract

Since the start of the coronavirus disease-2019 (COVID-19) pandemic, there has been interest in using wastewater monitoring as an approach for disease surveillance. A significant uncertainty that would improve the interpretation of wastewater monitoring data is the intensity and timing with which individuals shed RNA from severe acute respiratory syndrome coronavirus 2 (SARS-CoV-2) into wastewater. By combining wastewater and case surveillance data sets from a university campus during a period of heightened surveillance, we inferred that individual shedding of RNA into wastewater peaks on average 6 days (50% uncertainty interval (UI): 6–7; 95% UI: 4–8) following infection, and that wastewater measurements are highly overdispersed [negative binomial dispersion parameter, *k* = 0.39 (95% credible interval: 0.32–0.48)]. This limits the utility of wastewater surveillance as a leading indicator of secular trends in SARS-CoV-2 transmission during an epidemic, and implies that it could be most useful as an early warning of rising transmission in areas where transmission is low or clinical testing is delayed or of limited capacity.

## Introduction

Since the onset of the coronavirus disease-2019 (COVID-19) pandemic, there have been over 170 million known severe acute respiratory syndrome coronavirus 2 (SARS-CoV-2) infections [1]. From early on in the pandemic, reporting delays and changes in testing effort and capacity have made timely surveillance difficult [2–5]. This led to an interest in using the concentration of SARS-CoV-2 RNA in wastewater as a tool for COVID-19 surveillance and to monitor for secular trends. In the past, this type of surveillance has been used to provide early warnings of polio outbreaks [6] and to monitor for antimicrobial-resistant pathogens [7]. The initial hope that wastewater could be used as a leading indicator of SARS-CoV-2 transmission has led to mixed results [8, 9].

Understanding how temporal patterns in the incidence of new infections relates to the observed concentration of SARS-CoV-2 RNA in wastewater is key to interpreting wastewater surveillance data. These two quantities can be linked by the distribution, relative to the time of infection, of individual shedding of viral RNA into the wastewater system. This is analogous to the way in which the incidence of infection is linked to the timing of symptom onset by the incubation period in epidemiology. SARS-CoV-2 RNA has been observed in stool samples as early as a few days after hospital admission [10] and within a week of symptom onset [11], and as late as 5 weeks after respiratory samples are no longer positive for SARS-CoV-2 RNA [12]. The intensity with which SARS-CoV-2 is shed across this wide range of times relative to infection is presently unclear.

The primary objective of the present study was to use data from a COVID-19 outbreak on a university campus [13] to infer the shedding distribution of SARS-CoV-2 in wastewater relative to the time of infection. To do this, we utilised daily COVID-19 case notifications and daily measurements of SARS-CoV-2 RNA in the university sewage system. As approximately 85% of students live on campus [14], the sewage system is largely representative of the student body as a whole, which, when coupled with intense on-campus case surveillance and an outbreak with two distinct temporal peaks, makes this an ideal dataset for estimating the shedding distribution. As secondary objectives necessary to estimating the shedding distribution, we first estimated the time series of infections from the case notification time series, and described some pertinent aspects of the epidemic at the University.

## Methods

### Campus data

**Fig. 1.**
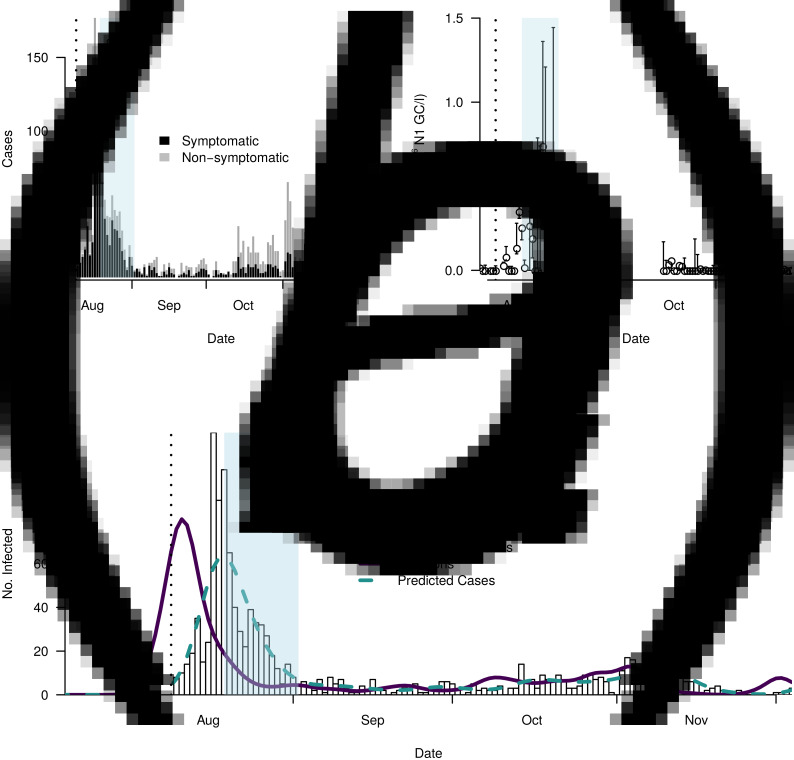
In all panels, the shaded blue region indicates a period of online instruction, where students remained on campus but did not have in person instruction, and the vertical dashed line indicates the start of classes (August 9); the majority of students arrived in the week preceding this date. (a) notified cases among students on the University campus in the fall semester, delineated by whether the case was found due to symptomatic screening or not. (b) the estimated timeseries of infections (purple line), alongside the actual (bars) and implied (green dashed line) distributions of symptomatic case notification dates. (c): wastewater measurements by date. Open circles indicate the daily mean of three recovery-corrected RNA measurements, and whiskers indicate the smallest and largest measurements on that day.

All case data come from the Fall semester in 2020 (10 August – 4 December inclusive). Approximately 7000 undergraduate students live on the University campus, about 85% of the total undergraduate population, although this may have been reduced during the pandemic. Early in the semester, the majority of cases were notified through screening of individuals with symptoms (Fig. 1a); there was initially no universal surveillance testing. The population used in this study was undergraduate students diagnosed through symptomatic testing (i.e., individuals who reported to the University health services with symptoms of COVID-19) via RT-PCR processed at an off-campus facility, in the Fall semester. This included those who lived both on- and off-campus, and excluded on-campus staff. We do not include those tested through asymptomatic screening or contact tracing, as these two routes were not consistently applied throughout the semester. Symptomatic screening proceeded throughout the semester, while a very limited amount of asymptomatic screening began on 21 August following the first peak, and proceeded through to the end of the semester. A recent study estimated the sensitivity and specificity of the RT-PCR tests to be 0.859 (95% CrI: 0.547–0.994) and 0.998 (95% CrI: 0.992–0.999) respectively [15]. Students arrived back on campus in time for the start of classes on August 9; most students arrived back in the final week before classes started, i.e. beginning on 2 August. Following high incidence in the first weeks back, the University underwent a 2-week period of online classes, starting on 19 August and ending on 2 September. Following notification, cases that lived on campus entered an isolation period, which typically lasted 10 days. While some of these cases were isolated in on-campus facilities, some cases were isolated off-campus, meaning they would not have been shedding into the campus wastewater system for that period. Additionally, on-campus contacts of notified cases were required to enter a period of quarantine of variable length dependent on test results [16].

### Wastewater data

From 3 August to 30 November 2020, 24-h time-based composite wastewater samples were collected each day from the manhole receiving all of the University wastewater. The wastewater system collects sewage from residence halls and facilities ranging from approximately 1600 to 5800 feet in the linear distance along the sewer lines and the average daily flow during the monitoring period was 3.29 million litres per day (s.d. ± 0.29). The low coefficient of variation (8%), coupled with the fact that storm water is collected via a separate system, suggests that the variation associated with wastewater flow rate would be small compared with other sources of variation. Sampling was interrupted from 1 September to 9 September due to a breakdown of the composite sampler. Full procedures for the processing of the wastewater samples has been detailed elsewhere [17]. Briefly, the composite sample was programmed to collect one 50 ml sample every hour, 24-h per day, throughout the sampling period. The wastewater composite sample from each day was mixed well and a 100 ml aliquot was removed, spiked with a process control (bovine respiratory syncytial virus; BRSV), which has been observed to mimic the recovery of SARS-CoV-2 from wastewater [18], and filtered using an electronegative membrane. After filtration, the membranes were aseptically rolled into bead tubes and homogenised using a bead beater, and the resulting liquid was extracted using a Qiagen PowerViral DNA/RNA kit. As detailed elsewhere [18], the resulting purified nucleic acids were assayed in triplicate for SARS-CoV-2 RNA using the N1 assay and for the process control RNA in single reactions via RT-ddPCR. During the period of 21 September to 11 October, Qiagen PowerViral DNA/RNA kits were not available and an alternative extraction method was used; however, the resulting RT-ddPCR data from this interval did not pass quality assurance protocols and were removed from the resulting dataset. Given the recovery of BRSV was previously observed to reflect that of SARS-CoV-2 in municipal wastewater (efficiency mean: 4.0% ± s.d.: 12%) [18], the SARS-CoV-2 RNA concentrations in wastewater were recovery-corrected to account for the measured process efficiency as suggested during a previous method comparison [19]. We calculated the cross-correlation of the recovery-corrected RNA concentration time series with the case notification time series at lags of up to 17 days (Fig. S2).

### Infection timing estimation

To estimate the incidence of new infections, we deconvolved the symptomatic case notification time series with the distribution of the time from symptom onset to testing. We estimated the distribution of time from infection to testing as the convolution of the incubation period and a delay from symptom onset to testing. We approximated the incubation period as a log-normal distribution with parameters *μ* = 1. 621 and *σ* = 0.418, according to Lauer *et al*. [20], and the delay between symptom onset and testing as a Poisson distribution (see Table 1 for a summary of all parameters used). In the baseline scenario, we used a mean delay from symptom onset to testing of 2 days, but also tried 0, 1 and 5 days in sensitivity analyses (Figs S5–S7). We then deconvolved this distribution from infection to testing from the case notification time series by maximum-likelihood deconvolution, using the backprojNP function in the R ‘surveillance’ package version 1.18.0 [21–23]. This algorithm seeks to estimate the infection time series with the highest likelihood of reproducing the observed case series, using knowledge about the distribution of the delay from infection to case notification. The algorithm works by proposing an initial distribution of infection times, and then sequentially adjusting this estimate to maximise the likelihood of reproducing the notified cases. It uses a Poisson likelihood for the number of infections in a day, and makes no assumption on the temporal pattern of infections. We set the smoothing parameter *k* = 10 and used default settings for all other parameters. Setting the smoothing parameter to a relatively high value such as this helps ensure that the algorithm captures the features of the infection time series without overfitting the noise in the case notification time series.

**Table 1.** Summary of parameter. Where the symbol column is empty, there was no symbol used for that parameter

Parameter name	Symbol	Value	Notes
Delay mean	*1/λ*	2 days	Assumed, and varied in sensitivity analysis to 0, 1 and 5 days
Incubation period meanlog	*Μ*	1.621	From Lauer *et al*. [20]
Incubation period sdlog	*Σ*	0.418	From Lauer *et al*. [20]
Smoothing parameter		10	Assumed
95% Limit of detection		220 N1 GC/l	
Quarantine length		10 days	Taken from University protocols [16]
Shedding distribution shape	*Α*	*Estimated*	
Shedding distribution rate	*Β*	*Estimated*	
Proportion of infections detected	*p_r_*	*Estimated*	Estimated in baseline. Set to 0.24 in a sensitivity analysis, as used by the CDC [24]
Negative binomial size	*R*	*Estimated*	
Shedding distribution scaling parameter	*Θ*	*Estimated*	

### Shedding distribution inference

We modelled the individual shedding distribution, *σ*(*t*), as a gamma distribution with the shape *α* and rate *β,* with the origin being the date of infection for that individual. We then adjusted this for entry and exit from isolation using the probability that the individual had entered isolation *t* days after infection, *p_enter_*(*t*), the probability they had exited isolation by *t* days after infection, *p_exit_*(*t*), the probability that they are reported, *p_r_* and the proportion of reported cases entering isolation on day *s* of the epidemic, *p_i_*(*s*). The parameter *p_enter_*(*t*) was given by the cumulative probability that the incubation period and delay from symptom onset to testing (described in the previous section) had been completed by day *t*, and *p_exit_*(*t*) was just this lagged by 10 days – i.e.
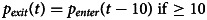

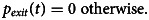


We estimated *p_i_*(*s*) by fitting a generalised additive model with a logit link to the proportion of infections entering isolation on day *s*, using the mgcv package (version 1.8–31) in R [25] (Fig. S1). We estimated the fraction of infections reported, *p_r_,* in the calibration (see below), assuming that it did not vary through time. In a sensitivity analysis, we set *p*_*r*_ = 0.24, to reflect the number of infections per reported case used by the CDC [24]. Given all these parameters, the individual shedding distribution adjusted for isolation was
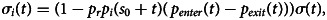
where *s*_0_ is the day of the epidemic on which the individual was infected. Note that as 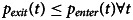, it follows that *σ*_*i*_(*t*) ≤ *σ*(*t*).

**Fig. 2.**
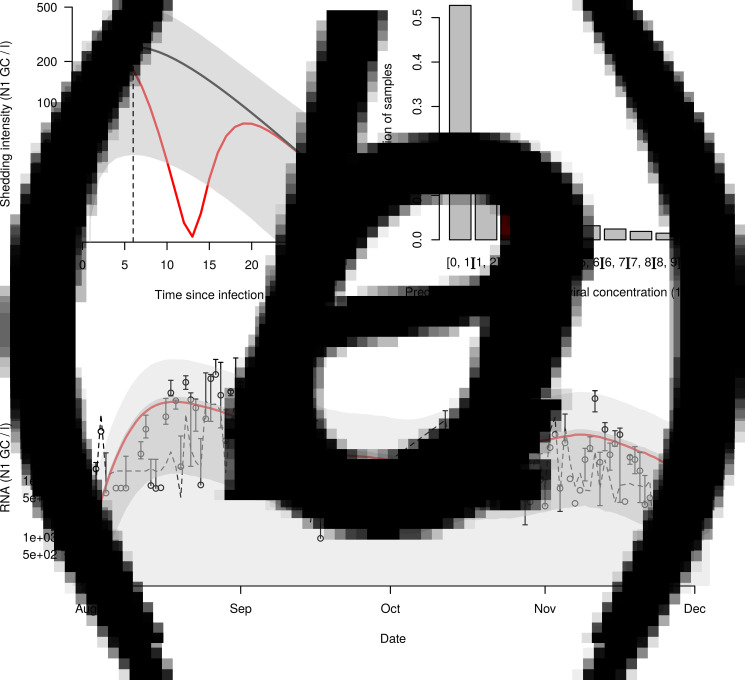
(a) Implied shedding distribution and 95% credible interval. The vertical dashed line indicates the day of peak shedding (8 days). The red line indicates the average shedding distribution of someone who enters isolation (b) Distribution of predicted daily recovery-corrected measurements at the peak intensity (August 16) implied by the negative binomial likelihood. The red shaded bar shows the range in which the predicted mean measurement fell (c) Mean predicted RNA concentration (red line), 95% prediction interval (light grey line). White open circles and whiskers indicate the daily mean measurements and minimum/maximum values respectively. The dashed line indicates the recovery-corrected 95% limit of detection. Data points below the 95% limit of detection are plotted as the midpoint of 0 and the 95% limit of detection for that day.

The number of new infections per day, *I*(*t*), was found by dividing the number of infected individuals on a given day that were ultimately reported, *I’*(*t*), by the proportion of infections that were reported, such that *I*(*t*) = *I*^′^(*t*)/*p*_*r*_. We then estimated the expected temporal pattern of SARS-CoV-2 RNA in the wastewater over time by summing the shedding distribution for each individual infected on a given day through time. We then multiplied this by a scaling constant, *θ*, to capture the magnitude of shedding. This yielded the reconstructed mean RNA concentration,
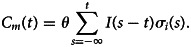


We fitted *C_m_*(*t*) to the observed RNA concentration in wastewater, *C_d_*(*t*), for each of the three daily replicate subsamples, using Markov chain Monte Carlo methods in the BayesianTools (version 0.1.7) R package [26], with a negative binomial likelihood, for which the dispersion parameter, *r,* was also fitted. When the RNA concentration in the wastewater was below the 95% limit of detection, we use the cumulative distribution function of the negative binomial distribution to determine the probability of observing data below that threshold. The baseline 95% limit of detection was 220 GC / l, which was then recovery-corrected, *D*_95_(*t*), and so varies through time (Fig. 2c). As a result, there were five free parameters: *α, β, θ, r,* and *p_r_*, with their likelihood given by
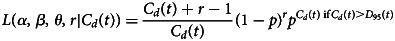

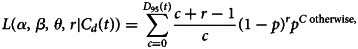
where *p* = *C*_*m*_/*C*_*m*_ + *r*. We used default settings from the BayesianTools package, and a uniform prior on all parameters with the following ranges: *α* ∈ (1, 10^3^), *β* ∈ (0, 10^3^), *θ* ∈ (0, 10^5^), *r* ∈ (0, 10^5^) and *p*_*r*_ ∈ (0, 1). We insisted that the shape parameter *α* was greater than 1 so that *σ*(0) *=* 0; i.e., people are not shedding at the moment they are infected. The MCMC chains and posterior parameter densities are shown in Fig. S9 and correlations between parameters are shown in Fig. S10. There were strong positive correlations between *α* and *β* and between *θ* and *p_r_*. In the former case, this was likely due to the fact that the mean of the gamma distribution was *α/β*. In the latter case, this was likely due to the fact that *θ* and *p_r_* appear as a ratio in the equation for *C_m_*(*t*), although *p_r_* does also appear in the isolation adjustment of the shedding distribution, so the two parameters could be separately identified. See Fig. S3 for examples of how different gamma distributions and delays from symptom onset to testing translate into different temporal patterns of viral RNA measurements in wastewater.

## Results

The majority of students arrived on campus in the week 3–9 August for the start of classes on 10 August. Soon after students arrived, there was a large number of reported COVID-19 cases among students, peaking at 177 cases reported on 17 August (Fig. 1a). In response to this, the University underwent a short period of online instruction from 19 August to 2 September, while students remained on campus. Case notifications declined thereafter and remained at a lower level, until rising again in October and November. In total, there were 2263 cases among students between the start and end of the semester (20 November). In the earlier part of the semester, the majority of notified cases were symptomatic when notified, though some asymptomatic and pre-symptomatic cases were detected through contact tracing and limited surveillance testing in specific groups, such as athletes. During the semester, surveillance testing capacity was substantially increased, and hence later in the semester, the majority of cases notified were not symptomatic at the time of detection (Fig. 1a). Throughout the semester, a proportion of students were isolated off campus for 10 days following a positive test result (Fig. S1). This proportion declined slightly over the semester, with about 25% of cases isolated off-campus over the entire semester.

From the time series of notified cases, we estimated the time series of infection incidence by deconvolving the case notifications with the distribution of the time between infection and testing (Fig. 2b) When we used this infection time series to project back the case-notification time series from which it was derived, we were able to recover the timing of peaks in notifications, although the height of the initial peak was underestimated. This underestimate in the peak is in part due to the smoothing step in the deconvolution algorithm, and in part due to the very steep nature of the peak, which is unable to be explained by the imposed distribution of time from infection to notification. This very steep peak may be simply due to chance, or due to a reporting artefact which meant notifications were clustered on a particular day. We estimate that a bare minority of transmission relating to the first large peak occurred prior to classes starting, as students were arriving back on campus, and that transmission was already rapidly declining when online classes began (Fig. 1b). Pre-arrival testing of all students, whether on-campus or not, ascertained 33 cases out of 11 836 tests.

Throughout this period, we collected one 24-h time-based composite sample per day, 7 days per week, from a manhole collecting all the wastewater produced on campus with no other inputs (storm water is collected via a separate system) and evaluated the concentration of SARS-CoV-2 RNA in these samples (Fig. 1c). As expected, the RNA concentration showed a similar temporal pattern to cases, though substantially more noisy. The cross-correlation function of RNA concentration and case notifications was highest at a lag of 7–9 days and was not significant at the 95% level at lags less than −1 days, implying that the wastewater was likely not a leading indicator of trends in case numbers in this context of relatively rapid case notifications (Fig. S2).

The shedding distribution we inferred rises rapidly following infection and through the incubation period, peaking at around 6 days (50% uncertainty interval (UI): 6–7; 95% UI: 4–8) following infection (Fig. 2a, Fig. S4A). The estimated genome concentration in a given sample was highly overdispersed, with only 28% of predicted samples being above the 95% limit of detection at the predicted peak SARS-CoV-2 RNA concentration (Fig. 2b, Fig. S4B). This is likely a combination of overdispersion in individual shedding and overdispersion from sampling due to the sewage not being well mixed at the point of sample collection. The maximum *a posteriori* estimate of the dispersion parameter was 0.393 (95% Credible Interval: 0.317–0.478).

To evaluate the fit of our inferred shedding distribution, we reconstructed the predicted concentration of SARS-CoV-2 in the wastewater (Fig. 2c, Fig. S4C). The mean predicted concentration captures the trends in viral concentration, though it peaks slightly too early – the peak in the mean predicted concentration occurred on 18 August, while the highest data point was recorded on 31 August and the highest daily mean and median on 27 August. The predicted time series were also very noisy, implying that it is necessary to average multiple replicates of each daily sample to gain an impression of temporal trends. This effect is partly due to variability in composite subsampling, and is likely to be heightened when the number of infections is small leading to more heterogeneity between replicates. The upper bound of our 95% prediction interval (i.e., the 97.5th percentile) was greater than 96.6% of measured values, and the upper bound of our 50% prediction interval (i.e., the 75th percentile) was greater than 81.6% of measured values. The Spearman's rank correlation coefficient of the mean predicted concentration and the mean daily estimated concentration was 0.51 (95% prediction interval: −0.01 to 0.54). When we increased or decreased the mean delay from symptom onset to testing, the predicted peak in the shedding distribution was a similar amount of time later or earlier respectively, but the overall shape of the distribution remained the same, and in particular its long tail (Figs S5–S7). The deviance information criterion (DIC; a measure of relative model quality, where lower values imply a better model) decreases as we increase the mean delay, with the lowest DIC occurring when the mean delay is 5 days. This implies that our baseline scenario of 2 days is perhaps more likely to give an underestimate of the timing of peak shedding than an overestimate. When we fix the proportion of infections that are detected to *p*_*r*_ = 0.24, we find that the timing of the peak is unaffected (Fig. S11). However, the mean magnitude of shedding is reduced, as this value of *p_r_* is less than our mean estimated value of 0.57. The uncertainty in the shedding intensity is also substantially reduced, reflecting that a large part of the uncertainty in our estimate of shedding intensity arises due to our lack of knowledge about the true number of infections.

## Discussion

The shedding distribution that we inferred peaks on average around 6 days after infection, before the onset of symptoms in 35% (95% credible interval (CrI): 15%–73%) of patients that become symptomatic [20]. Following this peak, the shedding intensity decays approximately exponentially, though at a lower rate than the initial increase. This slow decay means that, on average 77% (95% CrI: 44%–95%) of shedding occurs after the end of the incubation period. This early peak and slower exponential decay is broadly in line with measurements taken from stool samples by Wölfel *et al*. [11] and a meta-analysis of shedding in stool by Cevik *et al*. [27]. The early peak and slower decline indicate one reason why it can be difficult to use wastewater data as a leading indicator in certain circumstances: while some recent infections will be shedding, that signal may be masked by a large number of older infections. This is not a consequence of the sensitivity of the assays used to measure viral RNA in the wastewater, which have been shown to be remarkably sensitive [19, 28], but rather is due to the timing of that shedding. This is particularly the case in a period of declining or stable transmission, when the day-6 peak and the long tail in shedding suggest that much of the RNA measured may come from people infected some time ago, masking recent transmission. It is also important to consider that there is typically an additional delay in the analysis and reporting of wastewater data [29]. For these reasons, wastewater surveillance may be most useful to detect increased transmission in an area where there has recently been little or no transmission, or where there are greater testing delays and/or limited testing capacity [30]. One can observe this early warning in data from the end of July in our data set, where positive wastewater samples were recorded in advance of an increase in cases (Fig. 1a and c). If incidence stabilises at a relatively high level following an initial outbreak, the long tail in the shedding distribution implies that we may counterintuitively continue to see rising RNA concentration measurements for several weeks after case numbers have declined (see Fig. S8 for examplar incidence curves and the associated predicted mean wastewater measurements). Some studies have found that RNA concentrations do provide a short lead time on case data [8, 9]. This could be because the delay from infection to testing was longer in those studies than in ours, or because the shedding distribution peaks sooner in different age groups. Additionally, several authors have highlighted the complexity of the term ‘leading indicator’, as the lead time depends both on the type of application in question, and on the delay in processing and analysis of wastewater [29, 31].

A modelling study by Huisman *et al*., whose aim was to estimate the reproduction number over time (*R_t_*) directly from wastewater data, also estimated which shedding distribution minimised the error between estimates of *R_t_* based on wastewater and that based on cases [32]. Their inferred distribution had a much sharper peak, but also peaked around 6 days following infection. However, that study used data from larger populations and catchment areas and had a different and less direct calibration procedure, which may explain the discrepancy in shedding distribution shape. Using an approach originally described by Teunis *et al*., a study by Miura *et al*. attempted to estimate the shedding distribution directly from faecal samples rather than from case data [33, 34], They found a similarly prolonged period of shedding, albeit with an earlier peak. However, there were no faecal samples from the first 3 days following infection, and the highest concentrations occurred 9 days following infection, so their data are also consistent with a later peak in shedding. Data from studies like ours and that of Miura *et al*. highlight the importance of understanding the shedding distribution when seeking to estimate patterns of infection from wastewater samples. Another study (Schmitz *et al*. [35]) attempted to estimate individual shedding using data from a different university campus, finding that the mean shedding rate was 6.84 log_10_ N1 GC per gram of faeces. However, it is difficult to compare this directly to our estimates, as the Schmitz *et al*. study does not have a temporal component and is per gram of faeces, where ours is per litre of wastewater and agnostic to shedding route, i.e. it includes viral RNA shed through other routes [36]. While SARS-CoV-2 RNA load is usually dominated by shedding via faeces, shedding via saliva, sputum, or urine may make important contributions to the total load when the number of infections is low [36].

Our study has several limitations. First, we assumed that the distribution of testing delays was the same through time. In reality, there may have been longer delays earlier in the semester when testing capacity was lower and the number of cases was higher. Second, underreporting of cases means that our infection time series may not reflect the true magnitude of infections. If underreporting was constant through time, then we would still capture the temporal patterns in new infections, and hence the temporal distribution of shedding intensity would be correctly inferred, though if under-reporting increased over time this would lead to incorrect inference. We estimated the proportion of cases that were detected through symptomatic testing, assuming this was constant through time, to be 0.57 (95% CrI: 0.11–0.98). The high degree of uncertainty on this parameter estimate leads to great uncertainty in the magnitude of individual shedding. Third, our population was predominantly students, most of whom were between the ages of 18 and 22, and so extrapolations to other age groups may not be appropriate. For instance, in older populations or in the general population, there are likely to be fewer asymptomatic infections and consequently higher shedding rates. It is unclear whether or how the timing of shedding would differ in different age groups, but the comparability in the timing of our study and other studies not primarily focused on the 18–22 age group suggests that the timing of peak shedding may be robust to the age of the individual infected. There are also other factors that have changed since our study and which may also affect the shedding distribution, most importantly the emergence of new variants and vaccination. Finally, our inferred shedding distribution reflects the contribution each infected individual makes per litre of sampled wastewater, rather than per litre of wastewater produced by that individual. It, therefore, includes the effect of dilution. While this makes it difficult to estimate the magnitude of individual shedding, it should not affect our estimate of its temporal distribution.

In summary, we have estimated that infected individuals likely shed SARS-CoV-2 RNA into the wastewater for a prolonged period, peaking at around 6 days after infection, longer than the incubation period for COVID-19. This implies that wastewater data may be most useful to detect new outbreaks when incidence is low, when testing capacity is low, or when test results are substantially delayed. It also highlights that care must be taken when interpreting wastewater data during an ongoing period of high incidence.

## Supplementary material

For supplementary material accompanying this paper visit https://doi.org/10.1017/S0950268821002752. click here to view supplementary material

## Data and code availability

All underlying data and code to fit model and produce plots is available at https://github.com/scavany/wastewater_shedding_period
