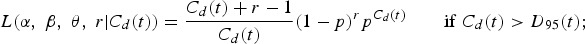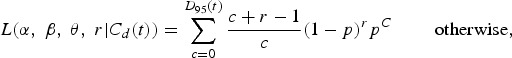# Inferring SARS-CoV-2 RNA shedding into wastewater relative to the time of infection – CORRIGENDUM

**DOI:** 10.1017/S0950268822000322

**Published:** 2022-03-04

**Authors:** Sean Cavany, Aaron Bivins, Zhenyu Wu, Devin North, Kyle Bibby, T. Alex Perkins

**Affiliations:** 1 Department of Biological Sciences, University of Notre Dame, Notre Dame, USA; 2 Department of Civil and Environmental Engineering and Earth Sciences, University of Notre Dame, Notre Dame, USA

The authors of the original publication would like to highlight some typographical errors from their article that may potentially cause some problems with interpreting parts of it.

The first error is in table 1: the parameter names are given in upper case, but they should be lower case. For instance, the Greek letters *μ, σ, α, β,* and *θ* should all be lower case, as should the *r* describing the negative binomial size. A corrected version of the table is shown below

**Table 1.** Summary of parameter. Where the symbol column is empty, there was no symbol used for that parameter

Parameter name	Symbol	Value	Notes
Delay mean	*1/λ*	2 days	Assumed, and varied in sensitivity analysis to 0, 1 and 5 days
Incubation period meanlog	*μ*	1.621	From Lauer *et al*. [20]
Incubation period sdlog	*σ*	0.418	From Lauer *et al*. [20]
Smoothing parameter		10	Assumed
95% Limit of detection		220 N1 GC/l	
Quarantine length		10 days	Taken from University protocols [16]
Shedding distribution shape	*α*	*Estimated*	
Shedding distribution rate	*β*	*Estimated*	
Proportion of infections detected	*p_r_*	*Estimated*	Estimated in baseline. Set to 0.24 in a sensitivity analysis, as used by the CDC [24]
Negative binomial size	*r*	*Estimated*	
Shedding distribution scaling parameter	*θ*	*Estimated*	

The second error concerns the likelihood equations (the equations immediately following the text “with their likelihood given by”). All of the text here is correct, but the last pieces of text in each equation is in the superscript, and should not be. For the first equation, this is everything including and following the word “if”, and for the second equation this is the word “otherwise,” Both of these should be rendered at the same level as the main part of the equation, and not in the superscript. The equations should appear as they do below.