# Supplementary material for: Inferring SARS-CoV-2 RNA shedding into wastewater relative to the time of infection
Source: Epidemiol Infect. 2022 Jan 7;150:e21. doi: 10.1017/S0950268821002752 (PMC8795777; doi:10.1017/S0950268821002752)
Supplement: Supplementary file 1 [file S0950268821002752sup001.docx]

**Supplementary figures**


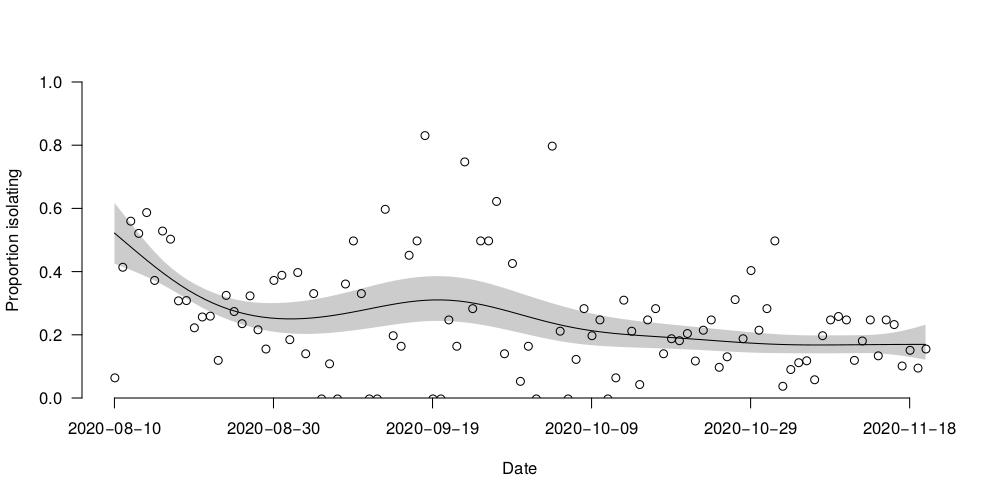


*Fig. S1: proportion of cases isolating in off-campus housing. Open circles indicate the proportion of notified cases that day that entered isolation that day. Black line and shaded region shows a generalized additive model with logit link fitted to this data, and the 95% confidence interval, respectively.*


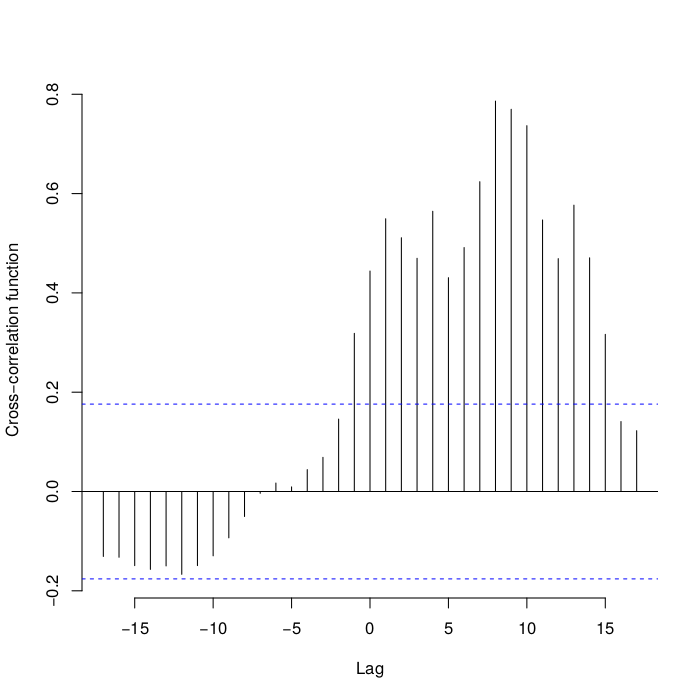
*Fig. S2: cross-correlation plot of case notifications and SARS-CoV-2 RNA concentration in the wastewater. Negative lags indicate that the RNA concentration is a leading indicator of changes in the case notification.*


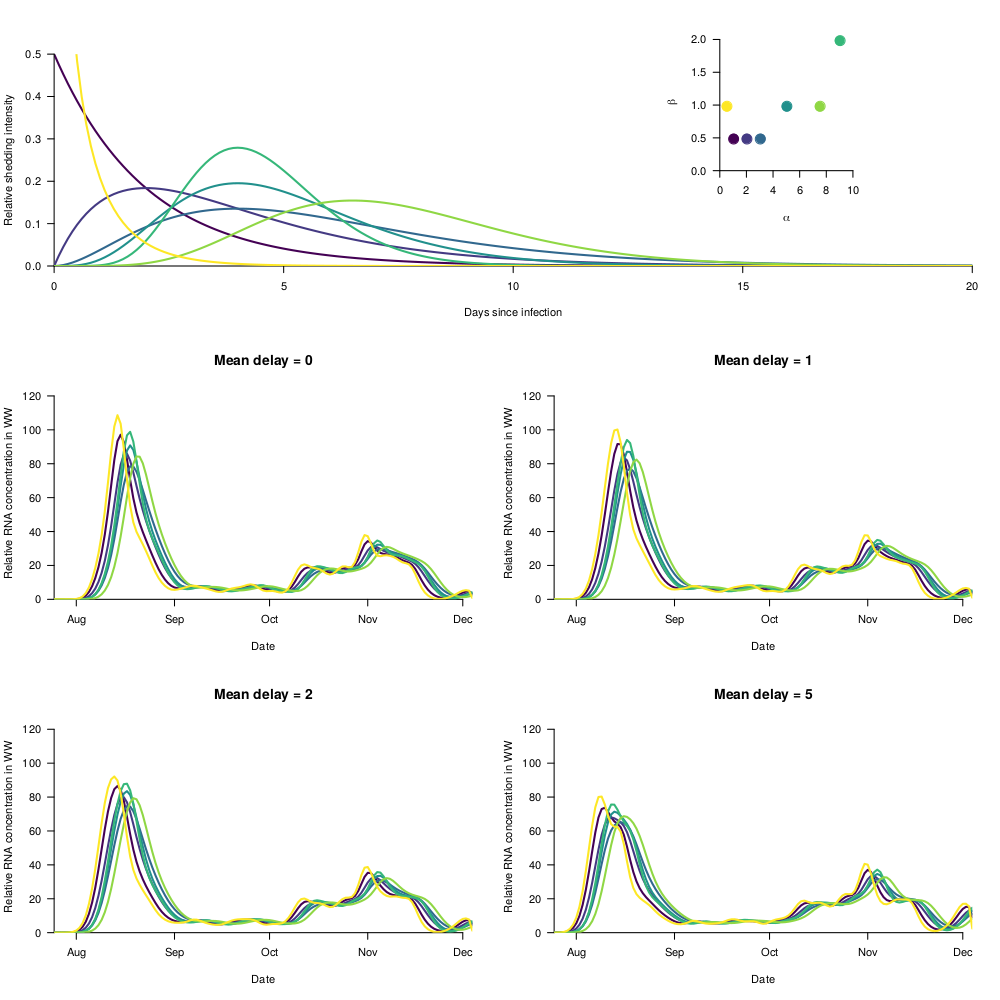


*Fig. S3: examples of possible shedding distributions. The top panel shows some example (arbitrary) shedding distributions. The bottom four panels show the implied temporal pattern of SARS-CoV-2 in the wastewater for each shedding distribution and for different delays*


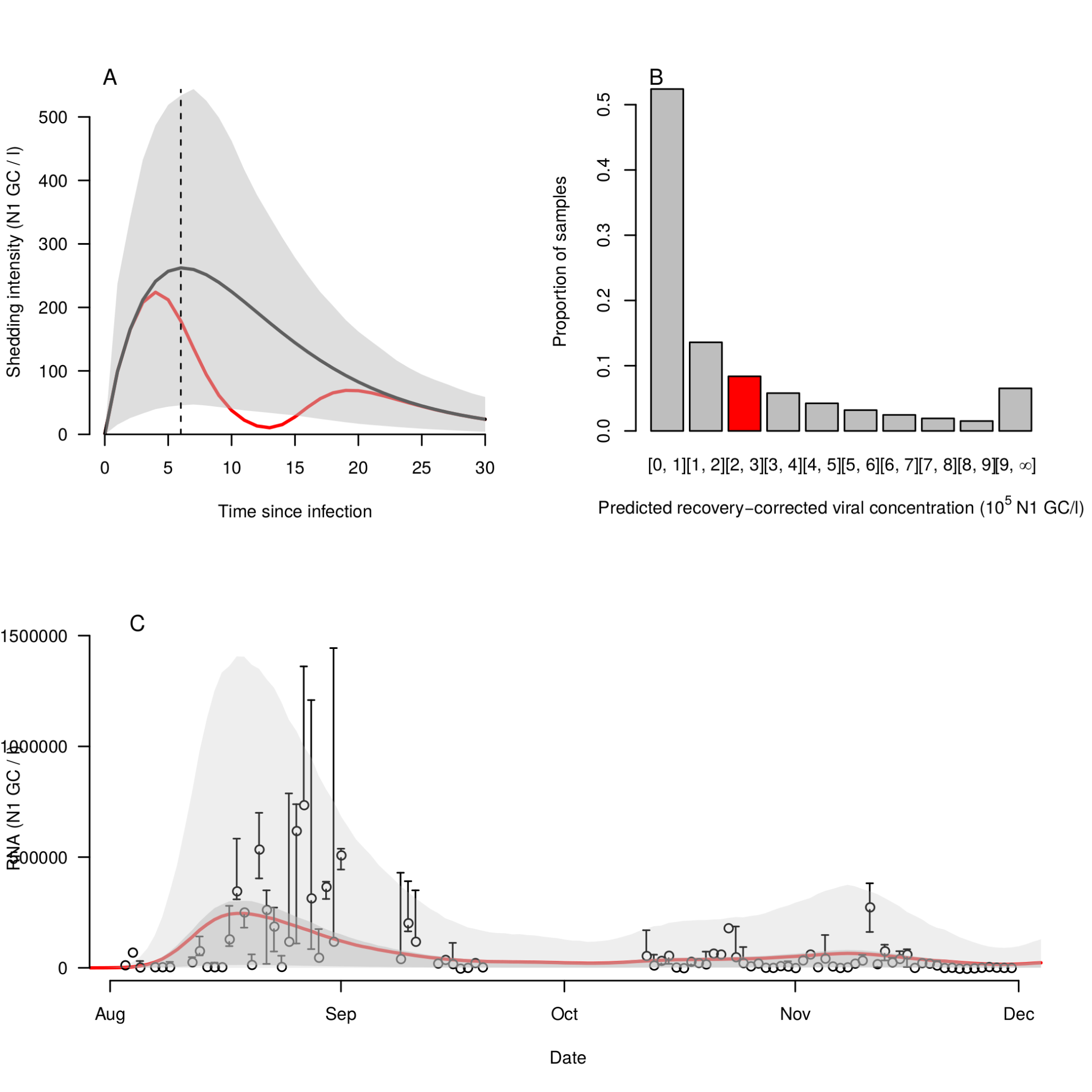
*Fig. S4: Shedding reconstruction with delay from symptom onset to test of 2, but on a natural rather than a log scale. Otherwise, as in Fig. 2.*


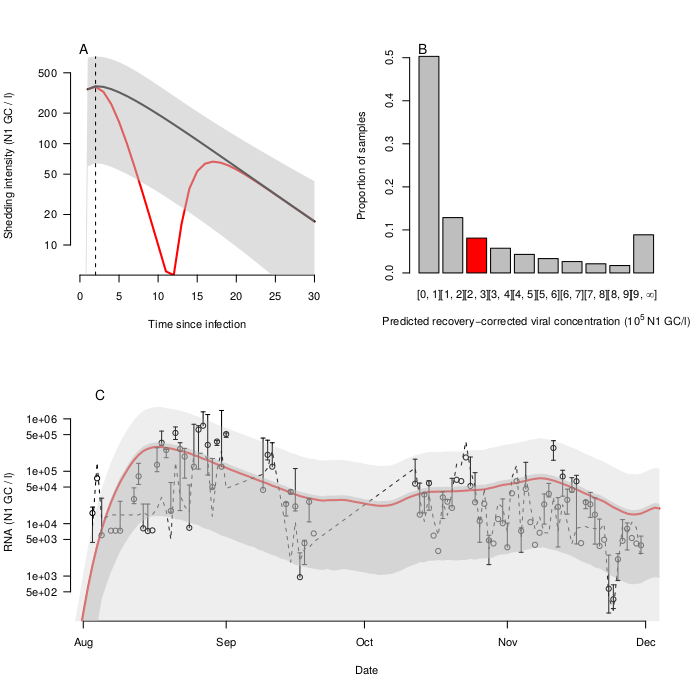


*Fig. S5: Shedding reconstruction with delay from symptom onset to test of 0. Otherwise, as in Fig. 2.*


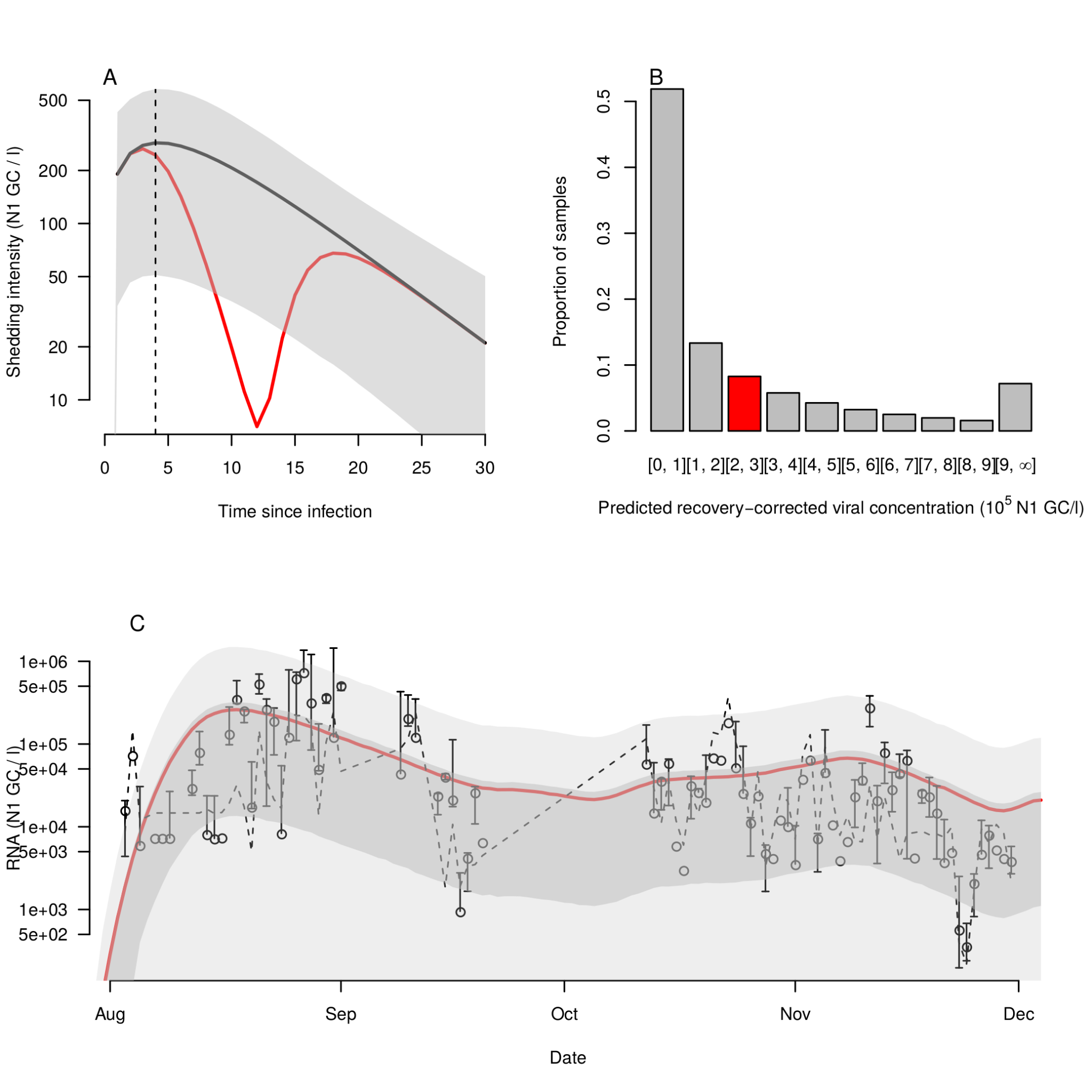


*Fig. S6: Shedding reconstruction with delay from symptom onset to test of 1 day. Otherwise, as in Fig. 2.*


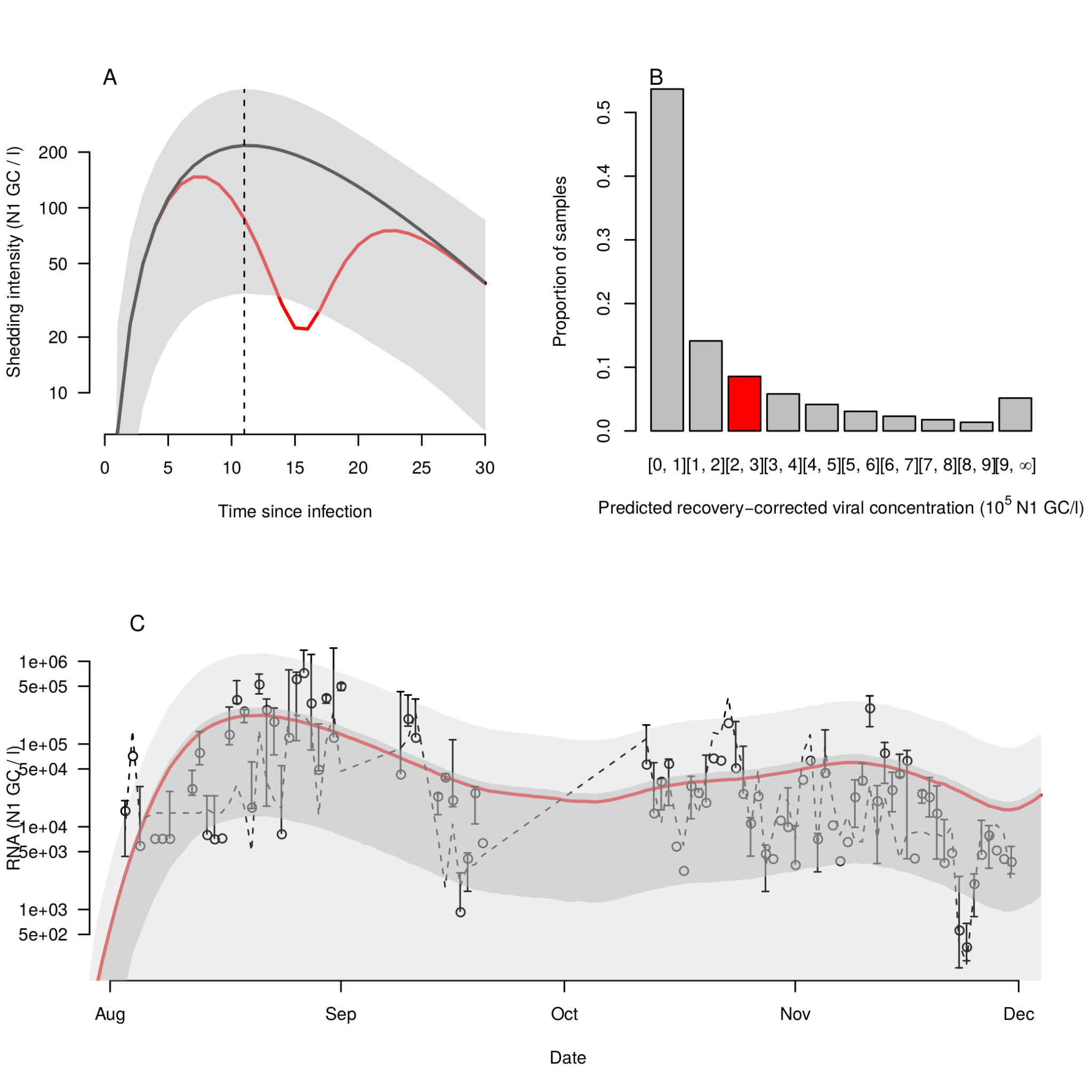


*Fig. S7: Shedding reconstruction with delay from symptom onset to test of 5 days. Otherwise, as in Fig. 2.*


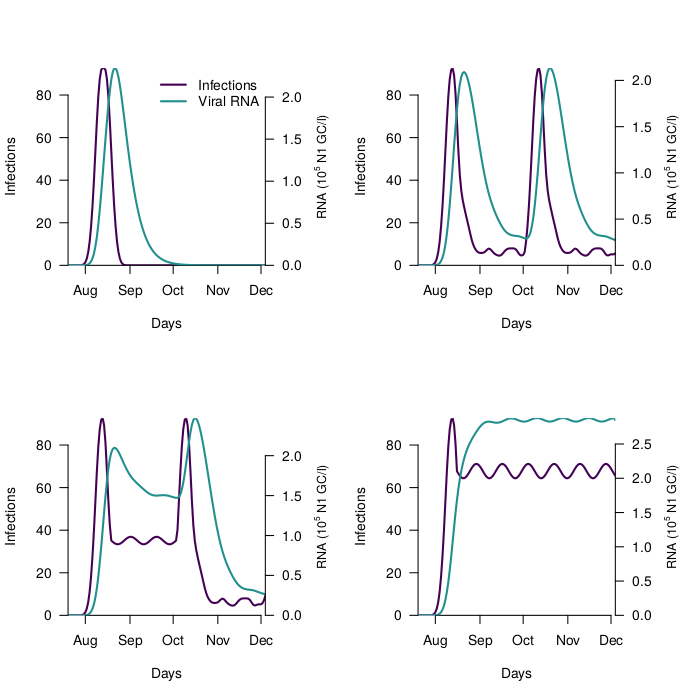


*Fig. S8: Example mean wastewater concentration timeseries for toy incidence curves, to show the relationship between potential incidence curves and wastewater data. In all panels, a mean delay of two days from symptom onset to test was used. Purple lines show synthetic examplar incidence curves, green lines show the implied mean RNA concentration in wastewater from these synthetic incidence curves.*


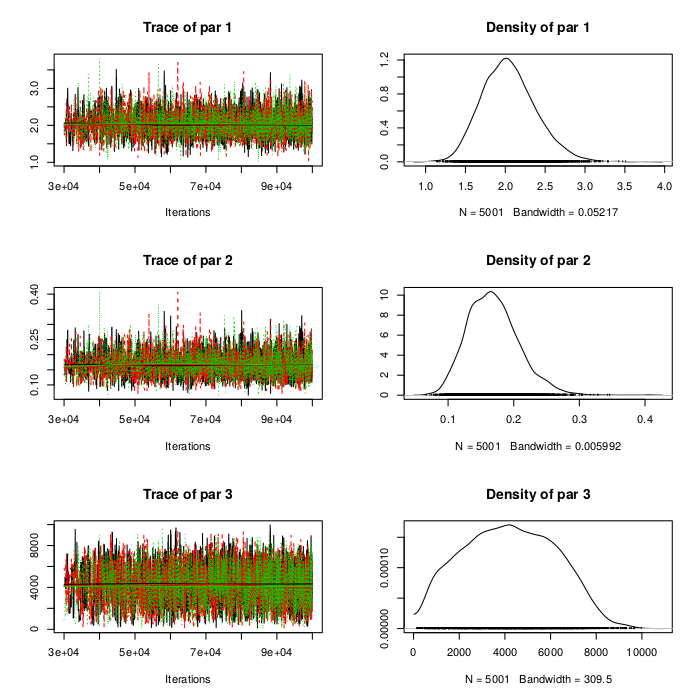


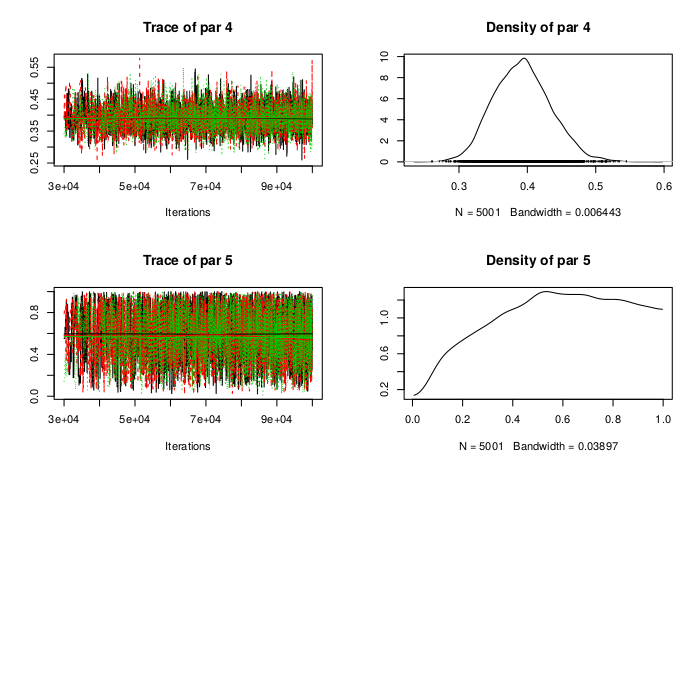


*Fig. S9: MCMC chains (left column) and posterior parameter densities (right column). par 1 is the gamma shape parameter (α), par 2 is the gamma rate parameter (β), par 3 is the scaling coefficient (θ), par 4 is the negative binomial size parameter (r), and par 5 is the proportion of infections reported (p_r_). There are three chains of 1x10^5^ iterations each, and the first 3x10^4^ iterations of each chain are burned.*


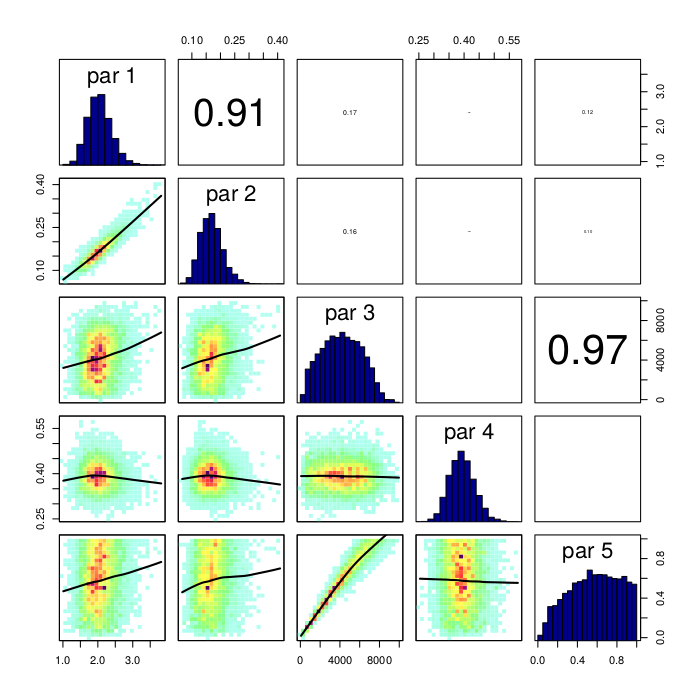


*Fig. S10: Posterior densities (diagonal), correlation density plots (lower left) and correlation coefficients between parameters (upper right). par 1 is the gamma shape parameter (α), par 2 is the gamma rate parameter (β), par 3 is the scaling coefficient (θ), par 4 is the negative binomial size parameter (r), and par 5 is the proportion of infections reported (p_r_).*


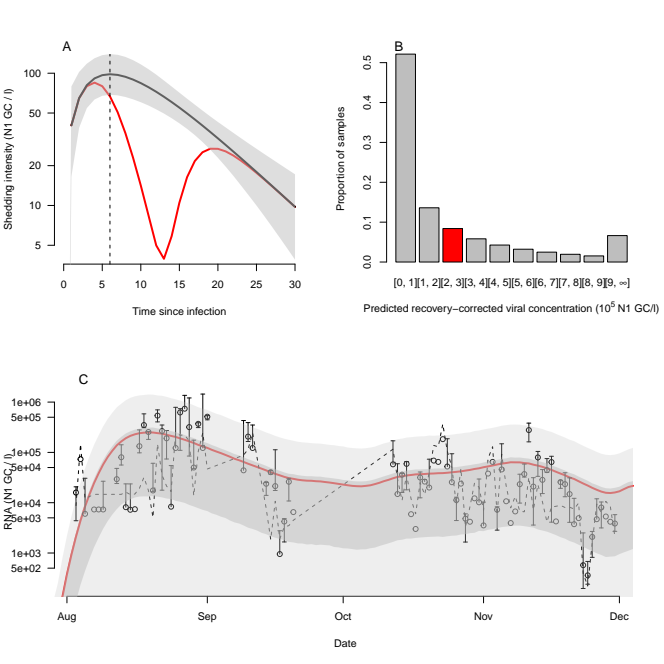
*Fig****.*** *S11: Shedding reconstruction with delay from symptom onset to test of 2 days and a fixed level of under reporting of p_r_ = 0.24. Otherwise, as in Fig****.*** *2.*

**Supplementary table**

*Table S1: Mean values and 95% credible intervals of fitted parameters and DIC for each fitted model. α is the gamma shape parameter, β is the gamma rate parameter, θ is the scaling coefficient, r is the negative binomial size parameter, and p_r_ is the proportion of infections reported.*

| **Testing delay** | **0 days** | **1 day** | **2 days** | **5 days** |
| --- | --- | --- | --- | --- |
| **α** | 1.31 [1.02 – 1.79] | 1.63 [1.15 - 2.24] | 2.05 [1.45 - 2.77] | 3.53 [2.53 - 4.72] |
| **β** | 0.143 [0.0901 – 0.223] | 0.151 [0.0908 - 0.235] | 0.169 [0.101 - 0.254] | 0.225 [0.143 - 0.325] |
| **θ** | 4,610 [807 - 8,480] | 4,320 [768 - 8,090] | 4,210 [716 - 7,900] | 4,010 [670 - 7,510] |
| **r** | 0.376 [0.304 - 0.457] | 0.386 [0.312 - 0.471] | 0.393 [0.317 - 0.478] | 0.408 [0.329 - 0.497] |
| **p_r_** | 0.586 [0.111 - 0.978] | 0.574 [0.111 - 0.976] | 0.573 [0.115 - 0.977] | 0.569 [0.104 - 0.974] |
| **DIC** | 3,755 | 3,750 | 3,747 | 3,739 |
